# Sexually dimorphic development of the mesolimbic dopamine system is associated with nuanced sensitivity to adolescent alcohol use

**DOI:** 10.3389/fnbeh.2023.1124979

**Published:** 2023-02-22

**Authors:** Ari M. Asarch, Lauren C. Kruse, Abigail G. Schindler, Paul E. M. Phillips, Jeremy J. Clark

**Affiliations:** ^1^Center for Neurobiology of Addiction, Pain & Emotion, University of Washington, Seattle, WA, United States; ^2^Department of Psychiatry & Behavioral Sciences, University of Washington, Seattle, WA, United States; ^3^Graduate Program in Neuroscience, University of Washington, Seattle, WA, United States; ^4^VA Puget Sound Health Care System, Seattle, WA, United States; ^5^Department of Pharmacology, University of Washington, Seattle, WA, United States

**Keywords:** adolescent alcohol use, decision making, dopamine, adolescent development, nucleus accumbens

## Abstract

Alcohol use remains a major public health concern and is especially prevalent during adolescence. Adolescent alcohol use has been linked to several behavioral abnormalities in later life, including increased risk taking and impulsivity. Accordingly, when modeled in animals, male rats that had moderate alcohol consumption during adolescence exhibit multiple effects in adulthood, including increased risk taking, altered incentive learning, and greater release of dopamine in the mesolimbic pathway. It has been proposed that alcohol arrests neural development, “locking in” adolescent physiological, and consequent behavioral, phenotypes. Here we examined the feasibility that the elevated dopamine levels following adolescent alcohol exposure are a “locked in” phenotype by testing mesolimbic dopamine release across adolescent development. We found that in male rats, dopamine release peaks in late adolescence, returning to lower levels in adulthood, consistent with the notion that high dopamine levels in adolescence-alcohol-exposed adults were due to arrested development. Surprisingly, dopamine release in females was stable across the tested developmental window. This result raised a quandary that arrested dopamine levels would not differ from normal development in females and, therefore, may not contribute to pathological behavior. However, the aforementioned findings related to risk-based decision-making have only been performed in male subjects. When we tested females that had undergone adolescent alcohol use, we found that neither risk attitude during probabilistic decision-making nor mesolimbic dopamine release was altered. These findings suggest that different developmental profiles of the mesolimbic dopamine system across sexes result in dimorphic susceptibility to alcohol-induced cognitive and motivational anomalies exposure.

## Introduction

Alcohol remains the most frequently used substance among adolescents, and in this age group, there is a prevalence of high levels of binge drinking (Lees et al., 2020). During adolescence, cortical and limbic regions undergo major plastic changes that are sensitive to the harmful effects of toxic substances like alcohol (Spear, 2000; Chambers et al., 2003; Crews et al., 2007, 2016; Bava and Tapert, 2010; Marinelli and McCutcheon, 2014) resulting in long-lasting behavioral changes (Casey and Jones, 2010). Specifically, adolescent alcohol use (AAU) is associated with later-life deficits in adaptive decision making, impulsivity, and reward valuation (Goudriaan et al., 2007; Johnson, 2008; Brevers et al., 2014) and so it has been proposed that AAU arrests neural development, locking in adolescent phenotypes (Spear, 2000; Crews et al., 2019). Some of these behavioral alterations have been modeled in rodents. For example, male rats with a history of AAU have higher risk attitudes when tested on probabilistic reward tasks (Nasrallah et al., 2009). Notably, the same level of alcohol exposure during adulthood did not produce a change in risk attitude (Schindler et al., 2014), supporting the notion that there is a unique window of vulnerability during adolescence. Commensurate to this behavioral change, increased mesolimbic dopamine signaling has been reported during reward-related behaviors (Nasrallah et al., 2011; Spoelder et al., 2015), and in response to physiological stimulation (Schindler et al., 2016; Kruse et al., 2017).

Based upon this premise, we hypothesized that dopamine transmission peaks during adolescence in normal development but, following AAU, remains high into adulthood, promoting risk taking (and potentially other impulsive behaviors). Therefore, we tested evoked mesolimbic dopamine release across developmental time points, using stimulation procedures that discern neuronal terminal or cell-body mechanisms of potential age differences.

## Methods

### Animal and housing

Sprague-Dawley rats (39 females, 32 males; Charles River, Hollister, CA, USA) began experimental procedures at Post Natal Day (PND) 25 (gavage) or 27 (gelatin) for animals exposed to alcohol, or PND 30, 50, or 120 for neurochemical experiments without alcohol exposure. Rats were housed in polycarbonate tubs on a 12-h light/dark cycle (lights on a 06:00) for one week before this date. Water and rodent chow (Teklad, Harlan, Kent, WA, USA) was available *ad libitum* except as noted. All work in this manuscript was approved by the Institutional Animal Care and Use Committee of the University of Washington.

### Alcohol preparation, administration, and withdrawal

Alcohol administration through the voluntary consumption of gelatin containing alcohol was presented to adolescent rats (PND 30–50) in a gel matrix consisting of distilled water (76.67%), Knox Gelatin (3.33%), polyose (10%), and 190 proof ethanol (10%), whereas the control gelatin contained distilled water in place of ethanol. Preparation was as described (Rowland et al., 2005; Nasrallah et al., 2011; Schindler et al., 2014). Gels were available 24 h a day with *ad libitum* access to food and water. Gel intake levels were measured daily and expressed in g/kg of body weight. All rats had access to only control gelatin for the first three days; after which rats were divided into either ethanol or control gelatin groups matched by weight and baseline intake for 20 days of assigned gelatin intake.

While this mode of administration produces enduring effects on cognition (Nasrallah et al., 2009, 2011; Schindler et al., 2014, 2016; Spoelder et al., 2015; Kruse et al., 2017), it does not achieve blood-alcohol concentrations that model heavy episodic drinking in adolescents. Therefore, we also utilized a second model of AAU that produces higher blood-alcohol concentrations (Crews et al., 2016). Adolescent intermittent ethanol administration via intragastric (IG), alcohol was presented to adolescent rats (PND 25–55) as a mixture of 190-proof ethanol and distilled water (16 g/kg, 20% ethanol, weight over volume). One cohort of rats received a single daily IG administration of ethanol and the other cohort received a single daily IG administration of distilled water (comparable volumes of water) on a 2-day on/off schedule Animals were then weighed and monitored daily.

For both IG and gelatin methods, after the last day of administration, the animals underwent three to four weeks of withdrawal and were monitored daily for withdrawal symptoms (e.g., seizures, weight loss, lack of grooming, and anxious behavior). It is important to mention that no overt signs of withdrawal were observed. Once the withdrawal period was completed, the rats began a food restriction diet of 90 ± 2% of their bodyweight and were exposed to 45 mg sucrose pellets (Bio-Serv, Frenchtown, NY) in their home cage to reduce neophobia. Additionally, prior to the start of the behavioral tasks, the rats underwent one magazine-training session in a standard operant chamber (Med Associates, St. Albans, VT) where they were given 15 min to consume 10 sucrose pellets in the magazine tray.

### Probabilistic decision-making task

Risk attitude was tested in female and male rats using a probabilistic decision-making task. After magazine training was completed, rats were trained on an operant fixed ratio (FR) schedule to a criterion of >23 presses out of 20 total trials where one pellet was delivered following the depression of the left or right lever. Rats then underwent auto-shaping, where the rats were required to first nose-poke the magazine tray to begin the trail where the intertrial interval was increased from 0 to 15 s, the time to perform the trail initiating the poke was decreased to 10 s, and the intertrial interval was increased to 30 s.

Detailed methods for these and the following tasks can be found in previous publications (Nasrallah et al., 2009, 2011; Clark et al., 2012; Schindler et al., 2014). During the task, rats were presented with two levers flanking the magazine tray where one lever represented the certain lever (low-risk) and the other the uncertain lever (high-risk). The low-risk lever was associated with a certain (1.00) delivery of two sucrose pellets and the uncertain lever was associated with the probabilistic (1.00, 0.75, 0.50, 0.25, and 0.00) delivery of four sucrose pellets. Each session consisted of 24 forced trials followed by 24 free choice trials where each probability presented on a different day decreased in descending order with an intertrial interval of 45 s. During the forced choice trials and following the trial initiation, a single lever would extend and the pressing of that lever resulted in the illumination of the tray light signaling the delivery of the associated reward based on the certainty of that lever and probability of that day; whereas following trial initiation during the free choice trials, both levers were extended with a total of 10 s for that rat to choose between the two levers.

After the probabilistic decision-making was completed, female control and ethanol rats underwent anesthetized surgeries with fast-scan cyclic voltammetry to measure pedunculopontine tegmental nucleus (PPT) and medial forebrain bundle (MFB) stimulated dopamine release in the nucleus accumbens (NAcc) as follows.

### Non-survival voltammetry surgeries

To test dopamine transmission across adolescent development, dopamine release was evoked by MFB or PPT stimulation and measured with FSCV during non-survival surgeries in females and males during early adolescence (PND 30–35), late adolescence (PND 50–55) or adulthood (PND 120–125). Female adult rats from control and gelatin decision-making behavioral groups were also tested this way.

Rats were anesthetized with a 1.5 g/kg urethane (i.p.) and head-fixed in a Kopf stereotaxic instrument. The skull was exposed and burr holes were drilled targeting the NAcc (relative to bregma: 1.3 mm anterior and 1.3 mm lateral, MFB (relative to bregma: 4.6 mm posterior and 0.8 mm lateral), and PPT (relative to bregma: 8.0 mm posterior and 2.0 mm lateral). Another burr hole was drilled on the contralateral side for placement of the reference electrode (Ag/AgCl). A carbon-fiber microelectrode was centered above the NAcc burr hole and lowered 6.8–7.2 mm ventral from the top of the brain.

On completion of the experiment, current was passed through the voltammetry electrode to produce a lesion to aid histological identification of the recording location. Animals were then sacrificed using phenytoin/pentobarbital (Bethanasia) and their brains were harvested for histological analysis of the recording and stimulating electrode placement. On analysis of these data using one-way analysis of variance (ANOVA), there were no significant differences in electrode placement between experimental groups. Specifically, no differences were observed (*p* < 0.05) between working electrode placement in the NAcc (dorsal/ventral: *F*_(1,61) group_ = 0.522; medial/lateral: *F*_(1,60) group_ = 0.9984; anterior/posterior: *F*_(1,61) group_ = 1.275), stimulating electrode placement MFB (dorsal/ventral: *F*_(1,37) group_ = 0.7424; medial/lateral: *F*_(1,37) group_ = 0.7336; anterior/posterior: *F*_(1,37) group_ = 0.8156) or stimulating electrode PPT (dorsal/ventral: *F*_1,44) group_ = 0.7081; medial/lateral: *F*_(1,44) group_ = 1.315; anterior/posterior: *F*_(1,44) group_ = 0.4166) between experimental groups.

### Fast-scan cyclic voltammetry recording

For recordings, a triangular waveform was applied to the carbon fiber starting at a potential of −0.4 V, ramping up to 1.3 V, and back down to −0.4 V (vs Ag/AgCl) at a rate of 400 V/s and a 10 Hz (held at −0.4 V between scans; Wanat et al., 2013; Schindler et al., 2016). A bipolar stimulating electrode was then incrementally lowered into either the PPT or MFB and NAcc dopamine release was evoked by electrical stimulation of the bipolar stimulating electrode at 60 pulses (p), 60 Hz, and 200 μA. Once maximum stimulated dopamine release was achieved, stimulations occurred at varying currents, pulses, and frequencies, and corresponding input-output curves were recorded.

For recording of the first input-output curve, the stimulation current was varied at 25, 50, 100, 150, 200, 300, and 400 μA respectively, while the pulse was held at 60 p and the frequency was held at 60 Hz. Next, the stimulation pulses were varied from 48, 42, 30, 18, 12, 6, and 3 p respectively, while the stimulation current was held at 400 μA and the frequency at 60 Hz. Stimulations and their corresponding recordings were performed with 5 min between each variation, including 5 min in between manipulation of pulse and current.

### Statistical analyses

All statistical analyses were conducted using Prism 6 (GraphPad).

Stimulated dopamine release was analyzed with two-way mixed-measures ANOVA with stimulation current or the number of pulses as a within-subject, and age or treatment as a between-subject factor. Behavioral data for decision-making sessions were analyzed using a two-way mixed-measures analysis of variance using probability as a within-subject and treatment as a between-subject factor. For intragastric alcohol administration, data were analyzed using three-way mixed-measures ANOVA with probability as a within-subject and treatment and sex as between-subject factors. All data are presented as mean ± SEM and threshold for statistical significance was set at *p* < 0.05 within correction for multiple comparisons as appropriate.

## Results

### Stimulated dopamine release across development

**Figure 1:**
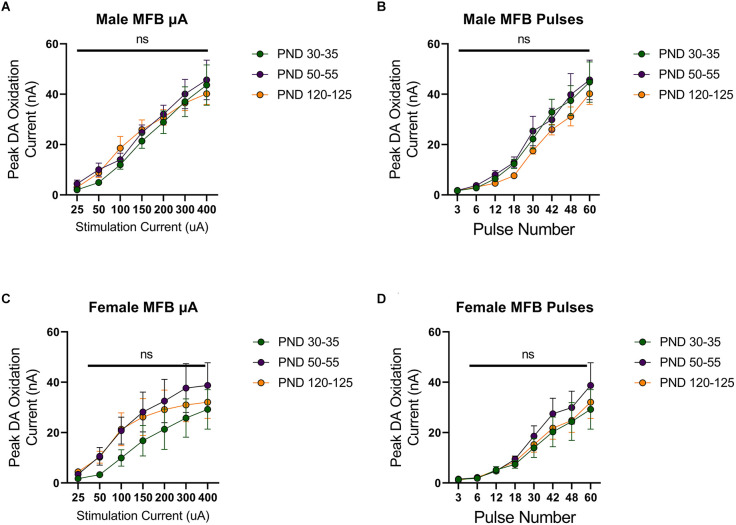
Peak dopamine (DA) oxidation current (nA) recordings in the nucleus accumbens (NAcc) over time after a single electrical stimulation of the medial forebrain bundle (MFB) in Males **(A,B)** and Females **(C,D)** averaged over varying stimulation currents **(A,C)** and pulses **(B,D)** at various ages. **(A)** Males’ PND 50–55 (*n* = 5) did not significantly differ from PND 30–35 (*n* = 4) and PND 120–125 (*n* = 4) in phasic DA release in the NAcc after stimulation of the MFB with increasing stimulation current. **(B)** Males’ PND 50–55 (*n* = 4) did not significantly differ from PND 30–35 (*n* = 4) and PND 120–125 (*n* = 4) in phasic DA release in the NAcc after stimulation of the MFB with increasing pulse number. **(C)** Females’ PND 50–55 (*n* = 6) did not significantly differ from PND 30–35 (*n* = 4) and PND 120–125 (*n* = 4) in phasic DA release in the NAcc after stimulation of the MFB with increasing stimulation current. **(D)** Females’ PND 50–55 (*n* = 6) did not significantly differ from PND 30–35 (*n* = 4) and PND 120–125 (*n* = 6) in phasic DA release in the NAcc after stimulation of the MFB with increasing pulse number. All data are presented as mean ± SEM for the peak DA oxidation current according to stimulation current (25, 50, 100, 150, 200, 300, and 400 nA) and pulse number (3, 6, 12, 18, 30, 42, 48, and 60). ns *p* > 0.05.

To investigate developmental changes in the excitability of mesolimbic dopamine neurons, we measured dopamine release in the NAcc in rats aged 30 (early adolescence), 50 (late adolescence), or 120 (adulthood) days postnatally. First, we evoked dopamine release with electrical stimulation of the MFB using a range of stimulation parameters. Dopamine release was reliably detected, with increasing peak dopamine concentration to higher simulation current or number of pulses in males (*F*_(1.576,15.76) current_ = 93.25, *p* < 0.0001, Figure 1A; *F*_(1.540,15.40) current_ = 80.27, *p* < 0.0001, Figure 1B) and females (*F*_(1.266,12.66) current_ = 31.79, *p* < 0.0001, Figure 1C; *F*_(1.942,19.42) current_ = 35.09, *p* < 0.0001, Figure 1D). However, these evoked dopamine-release profiles exhibited no significant differences across the tested developmental time points in males (current: *F*_(2,10) age_ = 0.2164, *p* > 0.05, *F*_(12,60) current × age_ = 0.5093, *p* > 0.05, Figure 1A; pulses: *F*_(2,10) age_ = 0.5778, *F*_(14,70) current × age_ = 0.3545, *p* > 0.05, Figure 1B) or females (current: *F*_(2,10) age_ = 0.7016, *F*_(12,60) current × age_ = 0.4490, *p* > 0.05, Figure 1C; pulses: *F*_(2,10) age_ = 0.4182, *F*_(14,70) current × age_ = 0.3201, *p* > 0.05, Figure 1D).

**Figure 2:**
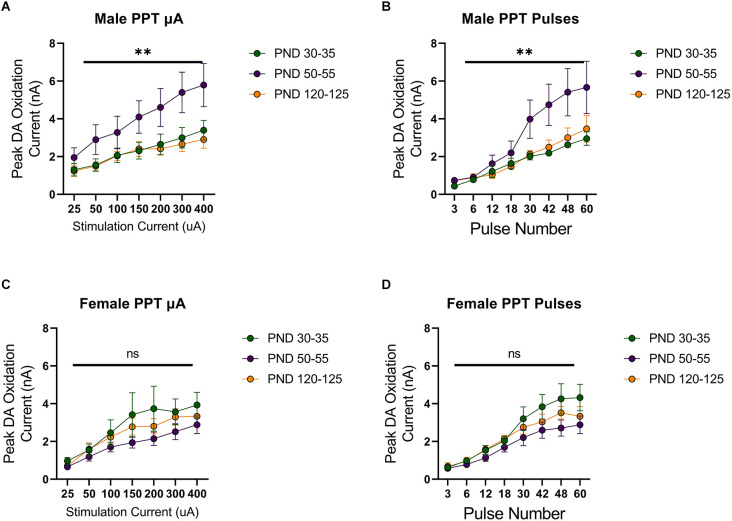
Peak DA oxidation current (nA) recordings in the NAcc over time after a single electrical stimulation of the pedunculopontine tegmental nucleus (PPT) in Males **(A,B)** and Females **(C,D)** averaged over varying stimulation current **(A,C)** and pulses **(B,D)** at various ages. **(A)** Males’ PND 50–55 (*n* = 6) significantly differed from PND 30–35 (*n* = 7) and PND 120–125 (*n* = 4) in phasic DA release in the NAcc after stimulation of the PPT with increasing stimulation current. **(B)** Males’ PND 50–55 (*n* = 5) significantly differed from PND 30–35 (*n* = 4) and PND 120–125 (*n* = 4) in phasic DA release in the NAcc after stimulation of the PPT with increasing pulse number. **(C)** Females’ PND 50–55 (*n* = 6) significantly differed from PND 30–35 (*n* = 4) and P120–125 (*n* = 4) in phasic DA release in the NAcc after stimulation of the PPT with increasing stimulation current. **(D)** Females’ PND 50–55 (*n* = 5) significantly differed from PND 30–35 (*n* = 4) and PND 120–125 (*n* = 4) in phasic DA release in the NAcc after stimulation of the PPT with increasing pulse number. All data are presented as mean ± SEM for the peak DA oxidation current according to stimulation current (25, 50, 100, 150, 200, 300, and 400 nA) and pulse number (3, 6, 12, 18, 30, 42, 48, and 60). ^**^p < 0.01 and ns *p* > 0.05.

**Figure 3:**
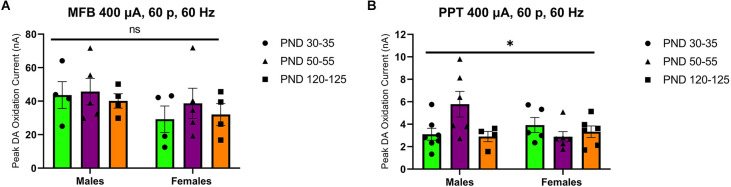
Peak DA oxidation current (nA) recordings in the NAcc for electrical 60-pulse stimulation (60 Hz, 400 μA) of the MFB **(A)** or PPT **(B)**. The pattern of NAcc dopamine release across development did not significantly differ between sexes (*n* = 13 per sex) for MFB stimulation **(A)** but was significant between females (*n* = 14) and males (*n* = 14) following PPT stimulation **(B)**. All data are presented as mean ± SEM. ^*^p < 0.05 and ns *p* > 0.05 (sex × age interaction).

Stimulation of the MFB activates ascending dopamine axons and provides an assessment of presynaptic (terminal) function in the control of dopamine release. To also assess the excitability of dopamine neurons to afferent input, we stimulated the PPT to evoke dopamine release by transsynaptic stimulation. PPT stimulation consistently increased dopamine release in a current and pulse-number sensitive manner in males (*F*_(1.557,21.79) current_ = 35.27, *p* < 0.0001, Figure 2A; *F*_(1.259,12.59) current_ = 29.33, *p* < 0.0001, Figure 2B) and females (*F*_(2.015,28.21) current_ = 30.13, *p* < 0.0001, Figure 2C; *F*_(1.362,17.70) current_ = 56.40, *p* < 0.0001, Figure 2D). Under these conditions, differences emerged across the developmental time points. Specifically, there was not a main effect of age on dopamine release in males (*F*_(2,14) age_ = 3.157, *p* > 0.05, Figure 2A; *F*_(2,10) age_ = 2.413, *p* > 0.05, Figure 2B) but the pattern of dopamine release for increasing simulation current differed across development (*F*_(12,78) current × age_ = 2.598, *p* < 0.01, Figure 2A) and pulse number (*F*_(14,70) current × age_ = 2.572, *p* < 0.01, Figure 2B), with the highest dopamine release during late adolescence (PND 50–55). However, there were no significant differences between developmental time points in females (current: *F*_(2,14) age_ = 1.249, *p* > 0.05, *F*_(12,84) current × age_ = 0.7699, *p* > 0.05, Figure 2C; pulses: *F*_(2,13) age_ = 1.823, *p* > 0.05, *F*_(14,91) current × age_ = 1.267, *p* > 0.05, Figure 2D). When directly comparing females and males, significant sex differences were not observed for MFB stimulation (*F*_(1,20) sex_ = 2.431, *p* > 0.05, *F*_(2,20) age_ = 0.4258, *p* > 0.05, *F*_(2,20) sex × age_ = 0.1330, *p* > 0.05, Figure 3A). For PPT stimulation, there were no significant main effects of sex (*F*_(1,28) sex_ = 0.9110, *p* > 0.05) or age (*F*_(2,28) age_ = 1.552, *p* > 0.05), but there was a significant interaction between these factors (*F*_(2,28) sex × age_ = 4.444, *p* < 0.05, Figure 3B). These data indicate sex differences in the developmental profile of dopamine neuron excitability which peaks in late adolescence in males but remains stable across the tested developmental time points in females.

### Effect of adolescent ethanol exposure on stimulated dopamine release in adulthood in female rats

**Figure 4:**
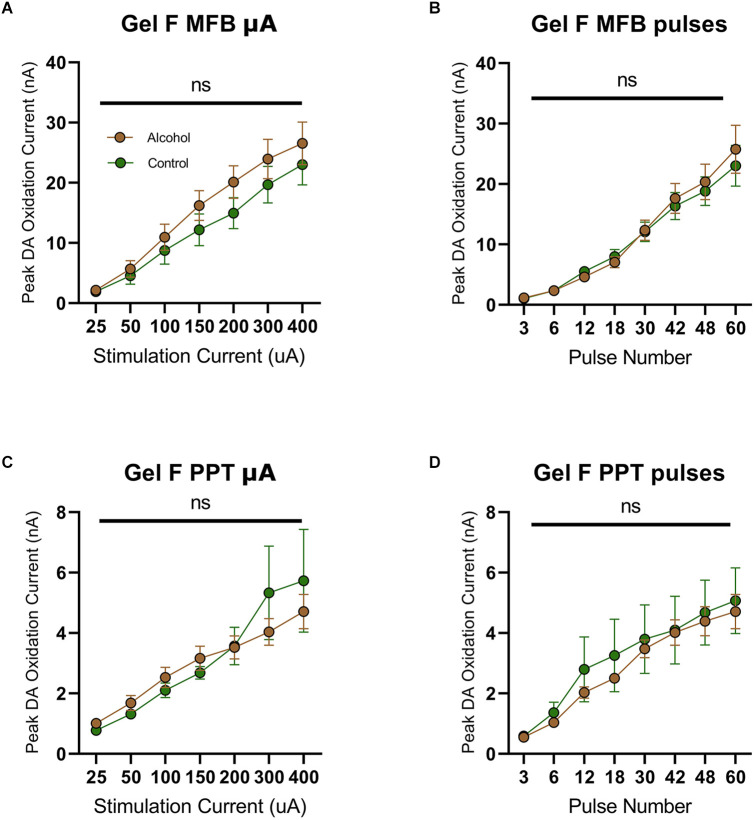
Peak DA oxidation current (nA) recordings in the NAcc over time after a single electrical stimulation of the MFB **(A,B)** and PPT **(C,D)** averaged over varying stimulation currents, pulses, and stimulation frequency for females. **(A)** Alcohol-exposed (*n* = 10) and control (*n* = 7) female animals did not significantly differ in phasic DA release in the NAcc after stimulation of the MFB with increasing current **(B)** Alcohol-exposed (*n* = 10) and control (*n* = 7) female animals did not significantly differ in phasic DA release in the NAcc after stimulation of the MFB with increasing pulse number. **(C)** Alcohol-exposed (*n* = 10) and control (*n* = 7) female animals did not significantly differ in phasic DA release in the NAcc after stimulation of the PPT with increasing stimulation current. **(D)** Alcohol-exposed (*n* = 10) and control (*n* = 7) female animals did not significantly differ in phasic DA release in the NAcc after stimulation of the PPT with increasing pulse number. All data are presented as mean ± SEM for the peak DA oxidation current according to stimulation current (25, 50, 100, 150, 200, 300, and 400 nA) and pulse number (3, 6, 12, 18, 30, 42, and 48). ns *p* > 0.05 for all.

Previously, we observed increased dopamine release during adulthood in male rats that consumed alcohol during adolescence (Schindler et al., 2016). This effect has been attributed to the notion of “arrested development” where adolescent phenotypes are locked in following AAU (Crews et al., 2019). However, based on the current findings, an interesting dilemma arises since dopamine release was not elevated in females during adolescence (Figure 3). Following this line of reasoning, we would not anticipate elevated dopamine release in adult females following AAU. To test this hypothesis, we measured phasic dopamine release in the NAcc of adult female rats with a history of AAU. Female rats were given access to gelatin containing 10% ethanol or vehicle 24 h a day for 20 continuous days (PND 30–50) and then dopamine release was assessed by MFB or PPT stimulation during adulthood (PND 90–110). Similar to experiments in male rats. stimulation of the MFB (*F*_(1.706,25.59) current_ = 51.09, *p* < 0.0001, Figure 4A; *F*_(1.091, 16.36) pulses_ = 70.77, *p* < 0.0001, Figure 4B) or PPT (*F*_(1.171,17.57) current_ = 22.71, *p* < 0.0001, Figure 4C; *F*_(2.111, 31.96) pulses_ = 36.96, *p* < 0.0001, Figure 4D) reliably elicited phasic dopamine release in the NAcc. However, MFB stimulation did not evoke a significantly higher dopamine release in female rats with a history of AAU when compared to controls in response to increasing stimulation current (*F*_(1,15) treatment_ = 1.031, *p* > 0.05, *F*_(6,90) current × treatment_ = 0.5739, *p* > 0.05, Figure 4A) or pulse number (*F*_(1,15) treatment_ = 0.05355, *p* > 0.05, *F*_(7,105) pulse × treatment_ = 0.3920, *p* > 0.05, Figure 4B). Likewise, there was no significant difference in electrically evoked dopamine release in the NAcc due to PPT stimulation in female rats who had a history of AAU in response to increasing stimulation current (*F*_(1,15) treatment_ = 0.03726, *p* > 0.05, *F*_(6,90) current × treatment_ = 1.203, *p* > 0.05, Figure 4C) or pulse number (*F*_(1,15) treatment_ = 0.2302, *p* > 0.05, *F*_(6,90) pulse × treatment_ = 0.2803, *p* > 0.05, Figure 4D). These data demonstrate that females exposed to AAU do not show a comparable increased release of PPT stimulated dopamine to that previously observed in males (Schindler et al., 2016).

### Effect of adolescent ethanol exposure on probabilistic decision-making in adult female rats

**Figure 5:**
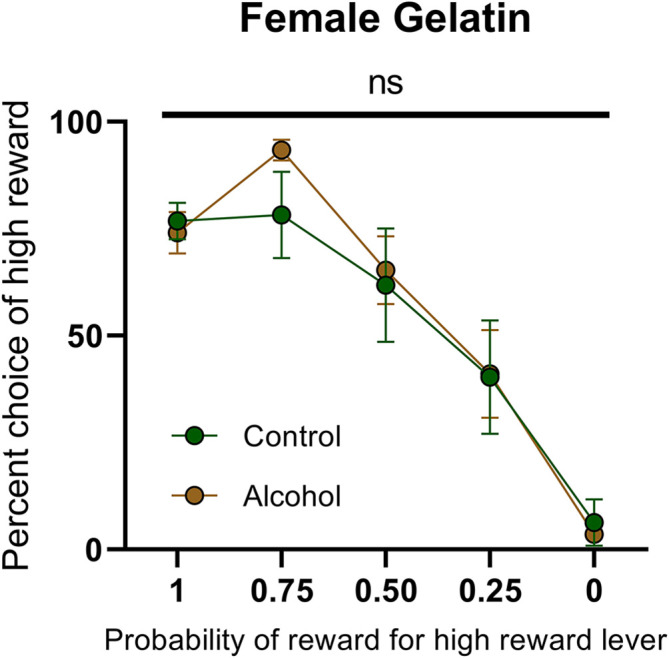
Psychometric function for choice over probability from alcohol-exposed and control animals. Alcohol-exposed (*n* = 8) and control (*n* = 8) animals did not differ in choice behavior over the probability of reward for high reward lever or in total reinforcements received. All data are presented as mean ± SEM for the percent of high choice reward according to the probability of reward for high reward lever of 1.00, 0.75, 0.50, 0.25, and 0.00. ns *p* > 0.05.

This failure to observe enduring changes in dopamine release following AAU in females provides an additional challenge on previous interpretations of AAU on cognition. The elevated dopamine in males is concomitant with an increased risk attitude during probabilistic decision making. However, the lack of elevated dopamine release in females questions whether the same behavioral changes would take place. Consequently, we trained female rats with AAU history (gelatin exposure described above) to perform probabilistic decision-making where animals choose between deterministic small rewards (two food pellets) and probabilistic large rewards (four food pellets). The probability of delivery of the large reward when chosen was systematically descended during each behavioral session (1.00, 0.75, 0.50, 0.25, 0.00). The frequency of choosing the large reward varied according to its probability of delivery (*F*_(4,55) probability_ = 31.40, *p* < 0.0001, Figure 5), but this pattern did not significantly differ between adult female rats with a history of AAU and their controls (*F*_(1,55) treatment_ = 0.2968, *p* > 0.05, *F*_(4,55) treatment × probability_ = 0.7999, *p* > 0.05, Figure 5). This result is surprising given that significant differences have consistently been observed across numerous cohorts of male rats (Nasrallah et al., 2009, 2011; Clark et al., 2012; Schindler et al., 2014, 2016) and, therefore, identifies a potentially important sexual dimorphism in the impact of AAU.

### Adolescent ethanol administration

**Figure 6:**
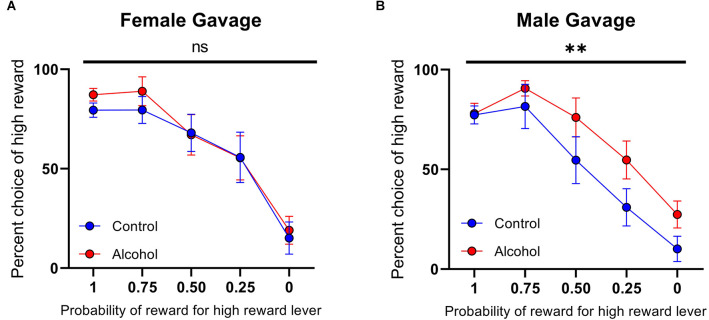
Psychometric functions for males and females receiving alcohol or vehicle via intragastric (IG). **(A)** Alcohol-exposed (*n* = 10) and control (*n* = 8) female animals did not differ in choice behavior over the probability of reward for high reward lever or in total reinforcements received. **(B)** Alcohol-exposed (*n* = 8) and control (*n* = 7) male animals did significantly differ in choice behavior over the probability of reward for high reward lever, but not in total reinforcements received. All data are presented as mean ± SEM for the percent of high choice reward according to the probability of reward for high reward lever of 1.00, 0.75, 0.50, 0.25, and 0.00. ^**^p > 0.01, ns *p* > 0.05.

Females that underwent the gelatin AAU model consumed 30.32 ± 1.96 g/kg/day (*n* = 12) of alcohol during adolescence. This value is considerably higher than male rats who underwent comparable training. For example, Schindler et al. (2016) reported 9.0 ± 1.2 g/kg/day in males (*t*_(28)_ = 9.833, *p* < 0.0001, unpaired *t-*test vs. females in the current study). Nonetheless, this model results in only moderate blood ethanol concentrations (Schindler et al., 2014) without reaching levels of heavy episodic drinking. Therefore, to test whether the lack of effect of AAU on risk taking in females was due to a dosing issue we next used a different model of alcohol administration that produces blood-alcohol concentrations at binge levels. Ethanol (20% w/v) was administered intermittently (cycles of two days on and two days off) via intragastric gavage between developmental days PND 25 to PND 55. Again, animals’ choices were sensitive to the probability of the high reward (females: *F*_(4,80) probability_ = 20.91, *p* < 0.0001, Figure 6A; males: *F*_(4,65) probability_ = 22.35, *p* < 0.0001, Figure 6B). Consistent with the gelatin model, the risk preference of female rats exposed to this treatment did not significantly differ from control rats on the probabilistic decision-making task (*F*_(1,80) treatment_ = 0.5446, *p* > 0.05, *F*_(4,80) probability × treatment_ = 0.1471, *p* > 0.05, Figure 6A). In contrast, male rats that received ethanol by gavage during adolescence exhibited a significantly different pattern of decision-making with increased preference for large probabilistic rewards over small deterministic rewards (*F*_(1,65) treatment_ = 7.764, *p* < 0.01, *F*_(4,65) probability × treatment_ = 0.6704, *p* > 0.05, Figure 6B). Accordingly, when females and males were compared directly, we found a significant effect of sex (*F*_(1,145) sex_ = 5.920, *p* < 0.05). These data demonstrate that, regardless of the method of ethanol administration and the amount received, AAU leads to risky decisions making in adult males, but not in females.

## Discussion

Here, we measured dopamine excitability across normal adolescent development. The experiments were designed to provide a platform to investigate the hypothesis that behavioral perturbations in adulthood arising from AAU are caused by alcohol-arresting post-adolescent neural development.

We observed a significant peak in dopamine neuronal excitability during the adolescent period in males. Therefore, if AAU arrests the development of this system then we would anticipate higher levels of dopamine during adulthood than in controls where dopamine excitability drops following adolescence. This prediction is consistent with our previous work demonstrating greater dopamine excitability during adulthood in animals that underwent AAU as compared to controls (Schindler et al., 2016). Moreover, the changes in dopamine across development were a result of mechanisms in the cell bodies rather than the terminals, as differences were not observed following axonal stimulation. Again, this change was consistent with altered dopamine release in adults following AAU (Schindler et al., 2016).

Conversely, in females, we did not observe changes in dopamine excitability across development. This lack of a peak in adolescent females was surprising, and seemingly refutes the idea that altered behavior in later life could be a result of arrested development of the mesolimbic dopamine system. However, this position assumes that AAU increases risk attitude during probabilistic decision-making in females like it does in males (Nasrallah et al., 2009, 2011; Clark et al., 2012; Schindler et al., 2014, 2016). In actual fact, the effect of AAU on probabilistic decision-making had never been tested in female rats. Indeed, in the current work, we failed to observe any difference in risk attitude between females that underwent AAU and controls. Because we were concerned that the lack of behavioral perturbation in females may be a dosing effect, then we repeated the experiment with an alternative method of alcohol administration that produced higher blood-alcohol concentrations. However, this approach again failed to produce altered risk-based decision-making in females despite being effective in male subjects. Therefore, females do not have elevated dopamine excitability in late adolescence; and alcohol use during this time does not produce elevated dopamine excitability, nor does it alter risk attitude, in adulthood. This pattern contrasts with males where dopamine excitability is elevated during late adolescence and drops in adulthood, but with alcohol use during this time, the elevated excitability is sustained into adulthood and animals exhibit a higher risk attitude during probabilistic (economic) decision making. While these concomitant neurochemical and behavioral findings are correlational, there has been a wealth of reports linking the NAcc (Kuhnen and Knutson, 2005; Zalocusky et al., 2016) and dopamine transmission (Fiorillo et al., 2003; Clark et al., 2012; Hart et al., 2015; Mortazavi et al., 2023) to risk taking, providing well-founded evidence for a causal relationship.

Our findings identify clear sex differences in the mesolimbic dopamine system during adolescent development and their correlation with the effect of AAU on later life physiology and behavior. However, it is not clear whether the dimorphic effects of alcohol are categorical, or whether they are quantitative effects. With regard to the dose of alcohol, females consumed a larger quantity of alcohol, during gelatin-based self-administration, than males in previous studies (Nasrallah et al., 2009, 2011; Clark et al., 2012; Schindler et al., 2014, 2016). Moreover, even with the much higher dosing achievable with gavage administration, females did not exhibit the altered risk taking observed in males undergoing the same alcohol dosing regimen. Therefore, it is unlikely that the sex differences are simply due to an insufficient dose of alcohol in the females. Another potential quantitative difference could be in the window of administration. Alcohol exposure took place over the same postnatal days in females and males, even though adolescent development is more advanced in females (Spear, 2000). However, alcohol administration by gavage was initiated earlier (PND 25) and extended to thirty days and still did not reveal an effect on risk-based decision-making in females. Of course, it is possible that this behavior in females may be susceptible to alcohol in the pre-adolescent postnatal period, but this developmental window was not tested in the current work. Nonetheless, given that males initiate alcohol use earlier on average than females—a trend that appears to be narrowing but is still significant in 2020 (White, 2020)—even if the female vulnerability is earlier in development, it would afford a distinct advantage to females at the population level.

Sex differences in the enduring effects of AAU have previously been observed (Bava and Tapert, 2010; Crews et al., 2016; Robinson et al., 2021; Maldonado-Devincci et al., 2022). Along with the current work, these data provide growing evidence for the nascent hypothesis that AAU can lock in adolescent cortical and limbic phenotypes and their downstream behaviors into adulthood, even when they are not comparable between sexes. This is particularly intriguing for traits that are sexually dimorphic in adolescence, but then normally converge in later life, since AAU could perpetuate these sex differences. Therefore, if the current findings translate to humans then the implication would be that females are protected against the effects of AAU on at least one cognitive process, specifically risk attitude.

## Data availability statement

The raw data supporting the conclusions of this article will be made available by the authors, without undue reservation.

## Ethics statement

The animal study was reviewed and approved by University of Washington Institutional Care and Use Committee.

## Author contributions

LK, AS, and JC conceived the studies. AA, LK, and AS performed the studies. AA analyzed the data. AA and PP wrote the manuscript. AA, LK, AS, and PP edited the manuscript. All authors contributed to the article and approved the submitted version.
